# Atrésie intestinale iléale: diagnostic anténatale et prise en charge

**DOI:** 10.11604/pamj.2016.24.240.9807

**Published:** 2016-07-15

**Authors:** Hanane Dhibou, Ahlam Bassir, Nadia Sami, Lahcen Boukhanni, Bouchra Fakhir, Hamid Asmouki, Abderraouf Soummani

**Affiliations:** 1 Service de Gynécologie Obstétrique, Pôle mère et enfant, CHU Mohamed VI, Marrakech, Maroc

**Keywords:** Atrésie intestinale, diagnostic anténatal, malformation congénitale, Ileal atresia, antenatal diagnosis, management

## Abstract

L’atrésie iléale est une malformation congénitale rare, elle constitue une faible part avec une incidence de 1 pour 5000 cas. Elle peut être suspectée et diagnostiqué échographiquement à la fin du deuxième et troisième trimestre. La concertation obstétrico-chirurgicale constitue ici la clé du succès. Eliminer une maladie générale à mauvais pronostic, lutter contre la prématurité et confier l’enfant immédiatement au chirurgien sont les objectifs principaux à réaliser. L'intervention chirurgicale va préciser le type de l'atrésie, son siège, son caractère unique ou multiple et sa longueur dont l’acte chirurgical dépend de l’étiologie. Il nous a paru intéressant de vous documenter un cas clinique d’atrésie iléale de diagnostic anténatal.

## Abstract (en)

Ileal atresia is a rare congenital malformation which affects a small percentage of the population, with an incidence of 1 in 5000 cases. It may be suspected and diagnosed by ultrasound at the end of the second and third trimester. Obstetrical and surgical consultation is the key to success here. Eliminating a systemic disease with poor prognosis, fighting to reduce premature births and immediately entrusting the child to the surgeon are the main objectives to achieve. During surgery, the surgeon will determine the type of atresia, its location, single or multiple areas of occlusion and its length; thus surgery depends on etiology. Our study reports an interesting clinical case of ileal atresia diagnosed antenatall.

## Introduction

L’atrésie iléale est une malformation congénitale rare, elle constitue une faible part de la pathologie traitée dans les centres de chirurgie pédiatrique avec une incidence de 1 pour 5000 cas [1]. Le diagnostic se fait en post natale mais la découverte anténatale est possible grâce à l’échographie obstétricale. La concertation obstétrico-chirurgicale constitue la clé du succès. Confier l’enfant immédiatement à la chirurgie pédiatre après un accouchement programmé permet de bien améliorer la prise en charge de cette pathologie. Il nous a paru intéressant de vous documenter un cas clinique d’atrésie iléale avec un diagnostic fait en anténatal et une revue de littérature.

## Patient et observation

**Figure 1:**
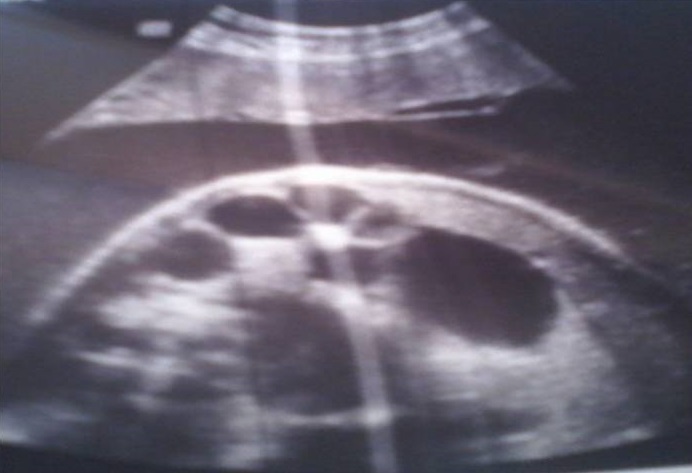
Aspect échographie d’une dilatation intestinale par atrésie iléale

**Figure 2:**
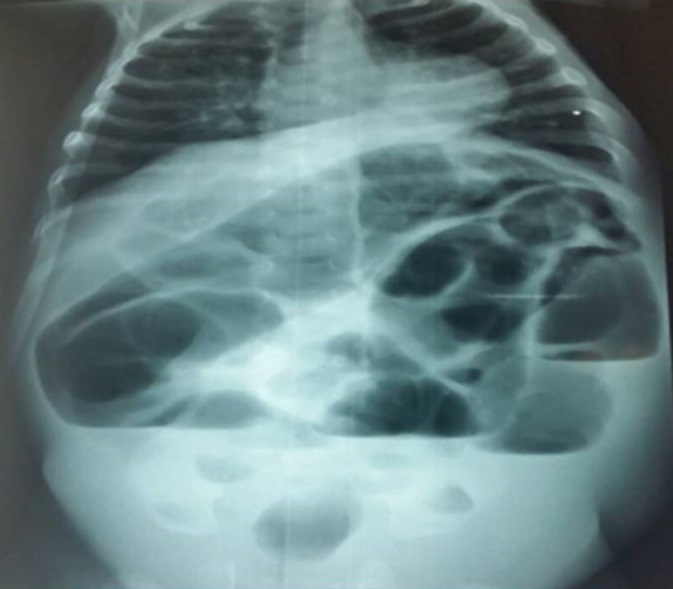
Une radiographie thoraco-abdominal post natale objectivant une distention grélique

**Figure 3:**
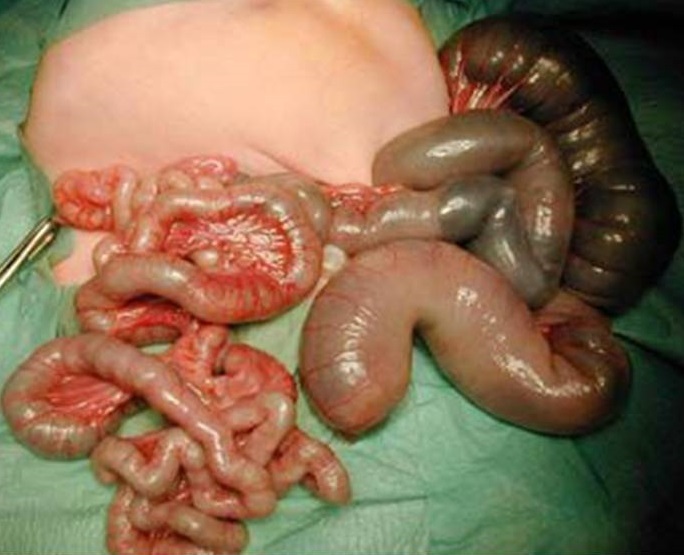
Image peropératoire révèlant une atrésie grélique incomplete

Nous rapportons le cas d’une parturiente âgée de 26 ans primigeste sans antécédents pathologique particulier. La grossesse était non suivie, une échographie obstétricale première réalisée à 35 SA a objectivée la présence dans l’abdomen de plusieurs dilatations anéchogènes à contours fins plus nombreuses, sans autres anomalies notables, y compris hydramnios, péritonite de méconium, gastroschisis et les défauts des autres organes (cœur, rein, foie, la tête et les os) (Figure 1). La couleur Doppler a montré une artère ombilicale normale. Devant ce tableau, le diagnostic d’atrésie iléale a été suspectée et la conduite obstétricale était une extraction à 39 SA par césarienne. La césarienne a donnée naissance d’un nouveau née de sexe féminin, Apgar 10/10. L’examen clinique à la naissance a révélé une distension abdominale manifeste, l’émission méconiale a été positive à la première heure de vie. La radiographie thoraco-abdominal face a montré des niveaux hydro-aréiques grêliques avec absence d’aération digestive (Figure 2). L’exploration chirurgicale à J3 de vie a révélée une atrésie grêlique iléale incomplète (Figure 3). Une stomie grêlique a été réalisée comme geste opératoire mais le nouveau né est décidé à J3 du post opératoire dans un tableau de choc septique.

## Discussion

**Figure 4:**
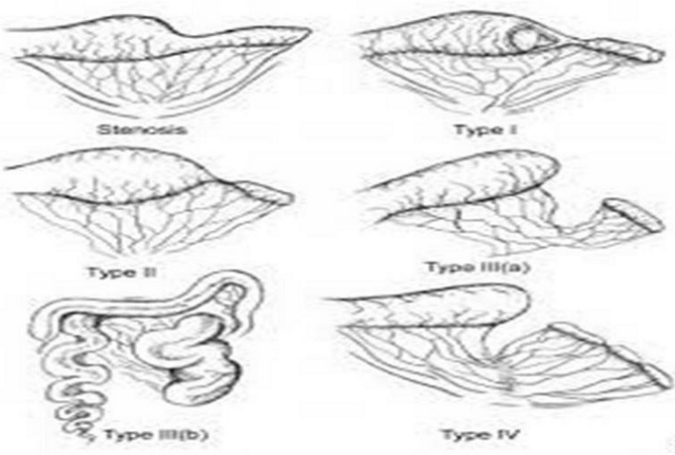
La classification des atrésies intestinales

Malformation congénitale rare, l’atrésie du grêle est une occlusion complète ou incomplète, selon qu’il y a ou non une solution de continuité et que la lumière est complètement interrompue ou pas. Parfois, les atrésies du grêle sont multiples, étagées tout au long de l’intestin (6 à 32% des cas selon les séries) [2]. Ces atrésies surviendraient vers la 12 SA, date de réintégration de l’anse intestinale primitive dans l’abdomen ou plus tard. La physiopathologie de l’atrésie intestinale étagée de survenu précoce de la vie fœtale est expliquée par l’hypothèse d’un trouble de reperméabilisation de l’intestin. Le mécanisme probable de l’atrésie grêlique unique succéderait à un épisode d’ischémie intestinale localisée, par atteinte d’une des branches de l’artère mésentérique supérieure soit due à un accident thrombotique primitif ou à un accident mécanique (volvulus, invagination, laparoschisis) aboutirait à l’interruption de la continuité de l’intestin [3]. L’incarcération du grêle dans une brèche mésentérique, un volvulus ou même une invagination anténatale [3] peuvent ainsi provoquer une atrésie du grêle par des phénomènes successifs de striction, de nécrose aseptique et de cicatrisation sténosante. L’association à l’atrésie d’une péritonite méconiale s’explique aussi par la perforation du grêle entraînée par la nécrose. La présence dans le segment d’aval de méconium et d’éléments épithéliaux résultant de la déglutition du fœtus sont des arguments en faveur d’un accident relativement tardif pendant la grossesse. L’atrésie du grêle peut être classée en quatre types: I atrésie en continuité, II atrésie avec interruption de continuité, III (a,b) atrésie en tire bouchon, IV atrésie multiple (Figure 4). L’atrésie de l’intestin peut être suspectée et diagnostiqué écho graphiquement à la fin du deuxième et troisième trimestre. Quand les boucles dilatées multiples d´intestins sont notées in utero qu´il va probablement être la sténose intestinale qui est en cause. L’aspect échographique objective une disparité de calibre importante, la portion dilatée en amont de la zone atrésie pouvant intéresser plusieurs anses et même l’angle duodénojéjunal, avec un diamètre qui peut atteindre cinq à dix fois le diamètre de l’intestin d’aval, lui-même minuscule [4].

Cette dilatation ajoutée à un retard de maturation des plexus mysentériques en aval de l’atrésie [5] explique en partie le dysfonctionnement postopératoire fréquent, qu’il faut chercher à prévenir par des artifices de résection et de modelage lors de l’intervention. Toute atrésie du grêle doit cependant faire rechercher une mucoviscidose surtout dans ses formes distales responsable de complications intestinales dans 13% des cas [6]. Une étude américaine a établi que le risque pour l’enfant présentant une atrésie du grêle d’être atteint de la mucoviscidose était 210 fois plus élevé que le risque pour la population générale [7]. En postnatal, elle se révèle par des vomissements bilieux associés à un météorisme abdominal et l´absence d´émission de méconium pour l’atrésie iléale complète, alors que l’émission méconiale de notre cas était précoce à H1 témoignant que l’atrésie était incomplète. L´ASP montre une dilatation du grêle avec présence de niveaux hydro-aériques centraux plus larges que hauts, sans aération colique. Les calcifications dans la cavité abdominale témoignent d´une péritonite méconiale liée à la perforation d´une anse. La recherche de malformations associées doit être faite dès que possible, la demande des examens étant orientée par la connaissance des associations les plus fréquentes pour chaque malformation. Ainsi une échographie rénale, cardiaque et médullaire est systématique dans toute occlusion haute néonatale, de même qu’un caryotype s’il existe des anomalies du faciès [8]. L’intérêt de diagnostic anténatal des atrésies intestinales réside dans la rapidité de la prise en charge chirurgicale dans le post natal immédiat vue qu’elle est une extrême urgence. La concertation obstétrico -chirurgicale constitue ici la clé du succès. Eliminer une maladie générale à mauvais pronostic, lutter contre la prématurité et confier l’enfant immédiatement au chirurgien sont les objectifs principaux à réaliser. L´intervention chirurgicale va préciser le type de l´atrésie, son siège, son caractère unique ou multiple et sa longueur. L’acte chirurgical dépend de l’étiologie: simple libération de brides de Ladd; effondrement d’un diaphragme muqueux, une résection de l´anse intestinale, un modelage et une anastomose termino-terminale permettent de rétablir la continuité du grêle [9]. Le pronostic est fonction du type d´atrésie, du siège et surtout de la longueur du grêle incriminé.

## Conclusion

Le diagnostic de l’atrésie iléale se fait en postnatal mais la découverte anténatale est possible grâce à l’échographie obstétricale réalisée en 2 et 3ème trimestre. L’intérêt de diagnostic anténatal de cette pathologie réside dans la rapidité de la prise en charge chirurgicale dans le post natal immédiat vue qu’elle est une extrême urgence.
